# Evaluating the effects of electronic health records system adoption on the performance of Malaysian health care providers

**DOI:** 10.1186/s12911-021-01447-4

**Published:** 2021-02-25

**Authors:** Mohd Idzwan Mohd Salleh, Rosni Abdullah, Nasriah Zakaria

**Affiliations:** 1 Faculty of Information Management, Universiti Teknologi MARA, Shah Alam, Selangor Malaysia; 2 School of Computer Sciences, Universiti Sains Malaysia, Gelugor, Pulau Pinang Malaysia; 3 Faculty of Medicine, Universiti Malaya, Kuala Lumpur, Malaysia

**Keywords:** Evaluation, Knowledge quality, Effective use, Clinician performance, Electronic health records, Total hospital information system, Partial least squares, Clinical practice guidelines, Consolidated framework for implementation research, Information management

## Abstract

**Background:**

The Ministry of Health of Malaysia has invested significant resources to implement an electronic health record (EHR) system to ensure the full automation of hospitals for coordinated care delivery. Thus, evaluating whether the system has been effectively utilized is necessary, particularly regarding how it predicts the post-implementation primary care providers’ performance impact.

**Methods:**

Convenience sampling was employed for data collection in three government hospitals for 7 months. A standardized effectiveness survey for EHR systems was administered to primary health care providers (specialists, medical officers, and nurses) as they participated in medical education programs. Empirical data were assessed by employing partial least squares-structural equation modeling for hypothesis testing.

**Results:**

The results demonstrated that knowledge quality had the highest score for predicting performance and had a large effect size, whereas system compatibility was the most substantial system quality component. The findings indicated that EHR systems supported the clinical tasks and workflows of care providers, which increased system quality, whereas the increased quality of knowledge improved user performance.

**Conclusion:**

Given these findings, knowledge quality and effective use should be incorporated into evaluating EHR system effectiveness in health institutions. Data mining features can be integrated into current systems for efficiently and systematically generating health populations and disease trend analysis, improving clinical knowledge of care providers, and increasing their productivity. The validated survey instrument can be further tested with empirical surveys in other public and private hospitals with different interoperable EHR systems.

## Background

### Adoption of the EHR system in Malaysia

Electronic health records (EHRs) are created from integrated health information systems via secured computer networks. These networks are available to authorized care providers for consultation and exchange purposes across health care settings [1]. In Malaysia, the EHR or Total Hospital Information System (THIS), is used to create EHRs to ensure full automation of hospitals and coordinated care delivery among various providers [2]. However, due to policy restrictions, hospitals in Malaysia have been implementing a non-shareable EHR system operated by a single or multiple authorized care providers within a particular facility [3]. In this system, the medical records of patients cannot be taken or used outside the hospital.

As developed in 1993, the EHR system was begun under the Sixth Malaysian Plan at the Selayang Hospital in 1999. It encompassed the total system framework, i.e., clinical, imaging, and administrative functions. New care facilities developed under the Seventh Malaysian Plan included HIS implementation starting with the primary system. While the EHR system was initially only for those hospitals with more than 450 beds, several Ministry of Health (MoH) hospitals across the country had begun to incorporate EHRs starting from the year 2000 onward [4, 5]. With the Malaysia HIE (myHIX) project initiated in 2008, these IT hospitals have been progressing towards implementing health information exchange (HIE) in participated MoH hospitals and clinics to enable the secure and smooth sharing of demographics and patient information, such as discharge summaries, referral letters, lab results, and imaging reports, through virtual private networks and later via cloud platforms.

The benefits of EHR systems are recognized mainly to support more excellent care, reduce medical resources, and improve clinical decisions [6]. However, without systematic evaluation, the system use could negatively affect job performance of clinical staff. In Malaysian tertiary referral centers, the use of clinical care IS was found to contradict doctors' workflows, their task complexities, and their work environments [7]. The doctors appeared to resist using the systems due to an inconvenient interface and functions, which have created many data entry mistakes and medication errors [8].

In one study, a group of researchers [9] identified HIS critical success factors by systematically reviewing pertinent studies published over the past 20 years (1996–2015). The review uncovered that the human factor was the most critical dimension in achieving HIS adoption success. Another study concluded that the successful application of HIS depends on how well the technology is implemented and how its use improves healthcare providers and hospitals [10]. In another research, Mohamadali and Zahari [11] recognized the challenges in the implementation of HIS in the Malaysian health industry, including (a) workflow disruptions with changing and complicated processes, (b) lengthy training procedures for learning HIS handling, (c) low computer hardware and network connectivity, and (d) loss of interest of physicians and nurses for using HIS due to lack of IT skills. All these factors were found to contribute to decreased adoption levels and productivity. Therefore, the “fit” among systems, records, technical support service, and knowledge is crucial in supporting the widespread acceptance of EHR systems and healthcare personnel [10, 12, 13].

The problems stated above give rise to the following question: to what extent do the quality of EHR systems, records, support service, and knowledge positively influence the effective use and performance of Malaysian health care providers? Existing studies in the local context have focused on adopting and accepting the EHR system and vaguely evaluated the providers' performance in using the systems [10–12, 14]. This gap necessitates developing a practical model that allows Malaysian clinicians to use EHR systems to effectively improve their work performance effectively. Accordingly, the present study aimed to evaluate several quality predictors' effects based on the effective use of EHR systems on healthcare providers' performance in a post-implementation stage.

### Theoretical gaps

Quantitative researchers have commonly adopted the DeLone and McLean (D&M) models to evaluate IS effectiveness [15, 16]. This evaluation framework has been generally applied to assess how several success factors can positively affect individuals and organizations. However, the D&M models appear to be common. Therefore, additional assessments are required to identify other potential factors that can positively influence clinicians' performance in using the EHR systems. An EHR system can manage and disseminate information to share knowledge and advance clinical research across multiple interoperable systems. Hence, a quality evaluation of IS should integrate knowledge quality for completion [17]. The use of the D&M model is also irrelevant due to the mandatory use of the EHR system [4, 18]. Therefore, the model must be revised with an improved measure for IS user performance when it is compulsory [2]*.* In measuring the success of IS, the D&M models delineate user satisfaction. However, a high relationship exists among system quality, information quality, and personal effect of user satisfaction construct [19], thus the low explanatory capability due to recurring measures [20]. Based on these justifications, user satisfaction is excluded in the performance measurement of care providers, but actual use will be improved with effective use.

### Research model

**Fig. 1:**
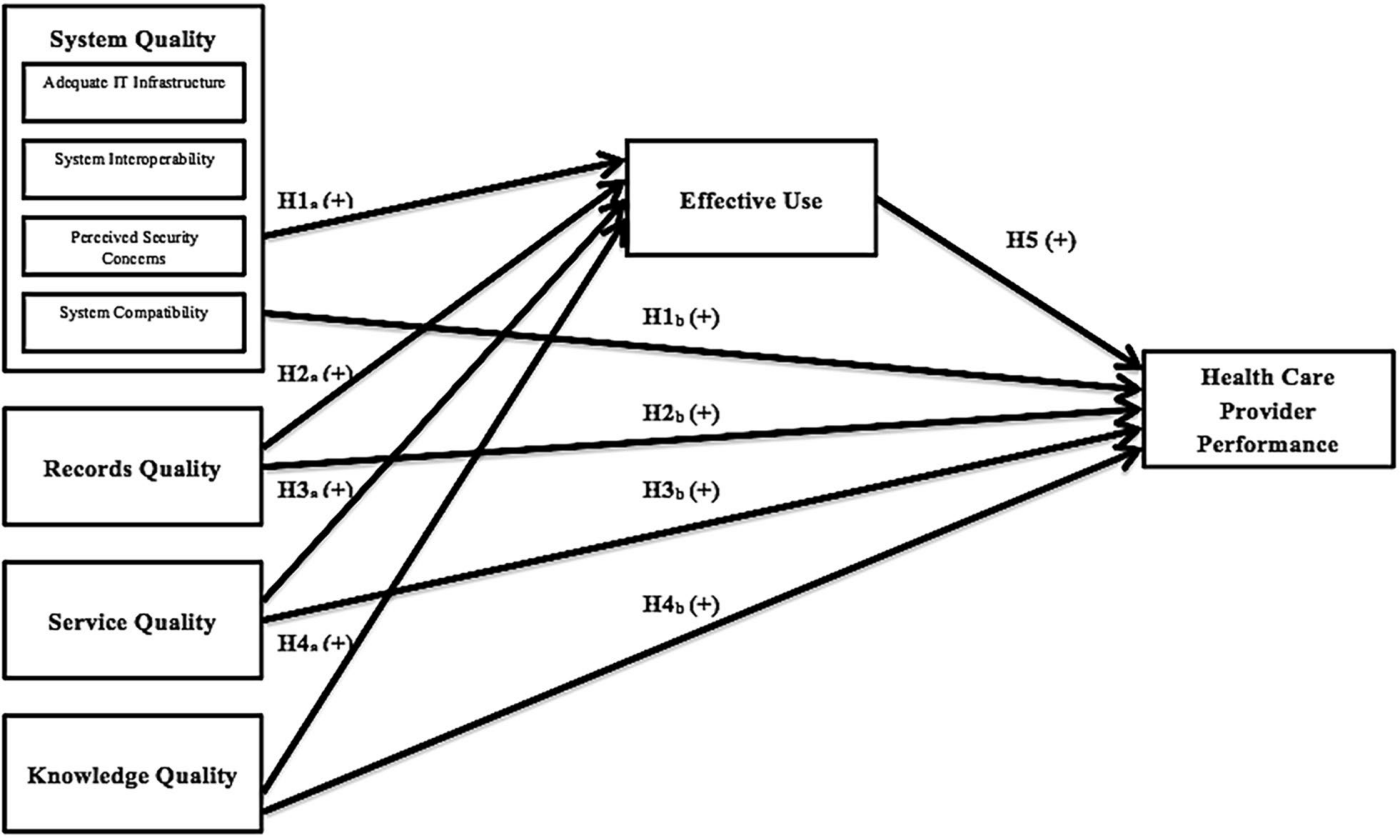
Proposed study model

Sets of relationships among exogenous, mediating, and endogenous constructs of the proposed study model are illustrated in Fig. 1. Each path possesses a positive hypothesized effect. The model comprises three exogenous constructs adopted from the DeLone and McLean (D&M) models, namely, system quality, record quality improvement through information quality replacement, service quality [15, 16], and knowledge quality (new construct), which are used as quality predictors. The D&M models are more appropriate for the problems being studied, the technical characteristics, the functionalities of local EHR systems, and prediction of the final performance outcome of end-users (health care providers) than other IT acceptance and user models, such as the unified theory of acceptance and use of technology and technology acceptance models.

The proposed study model evaluated the care provider effect at the individual level of analysis for those who deliver primary health care to patients by excluding the organizational impact framed in the conventional generic D&M Models. The organizational effect is more applicable in measuring the perceptions of IS success among diverse EHR stakeholders besides physicians and nurses. Hence, the effectiveness of the EHR system adoption is assumed when the primary care providers exhibit increased performance levels as predicted by the proposed predictors (system quality, record quality, service quality, knowledge quality, and effective use).

### Operationalization of study constructs

In a clinical setting, “system quality” refers to adequate IT infrastructure, system interoperability, perceived security concern, and compatibility of EHR systems with clinical tasks performed by care providers [21]. In this study, system quality is one of the quality factors used to measure care providers' effective use and performance. Second, record quality depends on timely access, consistency, standardization, accuracy, duplication prevention, and the completeness of EHRs generated from the system. Record term is preferred to information output because the former accurately describes the definition of EHRs as the repository of patient data available in digital format, which is stored, shared, secured, and accessed by authorized providers to support continuous and quality care [3, 22]. Examples of EHRs are patient treatment notes, images, laboratory test results, prescriptions, discharge summaries, patient histories, and medical reports [22]. Third, service quality denotes the quality of technical support delivered by EHR system vendors and internal IT personnel to measure effective use and clinician performance. As a newly proposed fourth exogenous construct, knowledge quality refers to the extent to which the health care providers can learn, create new knowledge, and apply what they have learned from an EHR system [17]. These can be done by consulting EHRs, clinician workflows, and best clinical practices, which can be applied to make the right decisions and solve patient problems. An enhanced effective use is identified as a mediator that enables clinicians to accomplish their clinical tasks without committing significant medical errors, misdiagnosis, or prescribing inaccurate medications.

### Study hypotheses

#### System quality

In the execution of clinical operations, EHRs rely on IT facilities, which influences the quality of patient care [23]. Doctors’ professional practices can be enhanced with excellent network connectivity [24]. In essence, interoperability means an EHR system's capability to access, use, transmit, and exchange EHRs from multiple integrated systems [25]. The interoperability of systems enables timely access to patient records for the benefits of cost reduction, speedy treatment, prevention of duplicated tests, and gradual improvement of doctor-patient relationships [26]. In a clinical setting, system security is HIS capability to protect the users and records from unauthorized access and against virus and bug threats [27]. These records should be acquired, stored, preserved, and used correctly and safely for high-standard care delivery [28]. Compatibility of technology with the work environment and organizational culture of health care providers is critical during system adoption [29]. The user will recognize the relative advantage of a system, whether it suits his/her job or style. In addition to task and workflow compatibility, a system design must also comply with standardized clinical practice guidelines (CPGs) [18]. Hence, the related hypotheses are as follows:

##### **H1**_**a**_

System quality has a positive effect on the effective use of EHR systems.

##### **H1**_**b**_

System quality has a positive effect on the performance of health care providers.

#### Record quality

EHR is a summarized version of patient health information compiled from the medical records [5]. Implementation of critical-care IS reduces documentation time and increases EHR quality and access time [30], positively affecting the system acceptance by doctors and nurses [7]. Similarly, physicians in intensive care units found that EHR use positively affects increased time spent on clinical review and documentation [31]. Thus, the related hypotheses are as follows:

##### **H2**_**a**_

Record quality has a positive effect on the effective use of EHR systems.

##### **H2**_**b**_

Record quality has a positive effect on the performance of health care providers.

#### Service quality

The positive attitude, performance, and satisfaction of clinical staff will improve when service providers deliver a high-quality support service [32]. Notably, the frequency of technical assistance visits will positively improve the use of an EHR system and physicians' work quality [33]. Hence, the related hypotheses are as follows:

##### **H3**_**a**_

Service quality has a positive effect on the effective use of EHR systems.

##### **H3**_**b**_

Service quality has a positive effect on the performance of health care providers.

#### Knowledge quality

EHRs primarily aims to integrate knowledge from patient health information in averting medical errors, thereby simplifying the analysis, presentation, and use of knowledge from EHRs. Clinical knowledge is generated from tacit knowledge (experiences or professional practices of care providers), converted into the explicit or documented form of CPGs, clinical workflows, and EHRs [17, 34]. An EHR system generates EHRs and stores CPGs and clinical workflows that contain knowledge [35], increasing its quality through sound clinical decisions and improved task productivity of clinicians [5, 12]. Hence, the related hypotheses are as follows:

##### **H4**_**a**_

Knowledge quality has a positive effect on the effective use of EHR systems.

##### **H4**_**b**_

Knowledge quality has a positive effect on the performance of health care providers.

#### Effective use

An integrated EHR system must enable physicians to complete their clinical tasks without making significant errors. Furthermore, its effective or extended use will positively affect physicians and medical practice [36]. The actual use of an EHR system that was previously measured on frequency or duration and extent of use has to be refined with effective use to achieve high individual and organization performance levels [37]. An effective system increases the needs, productivity, satisfaction, and motivation of clinicians to maximize the capabilities of the system [38]. Hence, the related hypothesis is as follows:

##### **H5**

The effective use of EHR systems has a positive effect on the performance of health care providers.

## Methods

### Study design

An EHR system–user evaluation survey was designed by selecting appropriate questions from past quantitative instruments designed based on the D&M models related to the study constructs and the local context of EHR system adoption. Responses were submitted through a 7-point Likert scale in which one represents "strongly disagree," and seven denotes "strongly agree." This scale offers the respondents considerable freedom of selection, as suggested by Redd et al. [39], and should thus be used in a survey instrument for improved reliability and validity after analysis. Before data collection, the questionnaire draft was further reviewed by IT officers from targeted hospitals because they had considerable experience conducting HIS satisfaction surveys. These officers then recommended that the number of questions is limited to fewer than 50 items to prevent inadequate response [5].

The pilot testing for the revised questionnaire was conducted among 100 medical professionals (five specialists, 55 medical officers, 20 assistant medical officers, and 20 nurses) at one general hospital with an EHR system in Selangor state. The result was further analyzed by Principal Component Analysis using the orthogonal rotation technique (Varimax) in IBM Statistical Package for the Social Sciences (SPSS). Specifically, for all measured constructs, Kaiser Meyer Olkin (KMO) measure of sampling adequacy was higher than 0.5, Bartlett's test of sphericity showed a significant value (*p* < 0.05), and the construct’s eigenvalue was larger than 1, which explained more than 50% of the variance in every construct with individual item loads higher than 0.4 [40], except for two System Quality items that were removed; therefore, the construct validity was confirmed. In total, 37 items were finalized for the field survey (Additional file 1: Survey Questionnaire).

By applying Faul et al.’s [41] guideline, a priori analysis was executed in G*Power 3.1 to compute the required sample size for the field study. The recommended samples were *N* = 146 (*f*^2^ = 0.15 [medium effect], *α* = 0.05, latent constructs = 6) to ensure the power of 0.95 at 5% level of statistical significance. Hence, a total sample of 438 was required to gather data from the three hospitals.

### Unit of analysis

The samples consisted of primary health care providers (specialists, medical officers, nurses) who were directly engaged in inpatient care from admission to discharge but excluding pharmacists, radiologists, and laboratory technologists [7, 12]. In particular, this study only focused on the use of major clinical functionalities such as admission, clinical care, EHR documentation, discharge, transfer, referrals, and deceased management of patients executed in five integrated system modules (patient, pharmacy, laboratory, radiology, operating theatre) by specialists, medical officers, and nurses. Hospital management, sponsors, administrative staff, billing team, system developers, vendors, or contractors, who were also EHR stakeholders, were not engaged. They typically used the EHR systems to perform administrative and non-clinical operations and positively affect the study validity [7, 10, 42].

### Data collection

Convenience sampling was employed to collect the data due to the specialists and medical officers' hectic schedules in the busy hospital environment, limiting random sampling. Upon receiving approval from the Medical Research and Ethics Committee (MREC), the survey questionnaire was administered (a) to the target samples during the continuing medical education (CME) programs for specialists, medical officers, and assistant medical officers, and (b) to the continuing nursing education (CNE) programs for nurses organized in different government hospitals that were implementing multiple EHR system packages with similar clinical functionalities. In the field survey, sample data were gathered from three respective MOH hospitals (a) with more than 500 patient beds and (b) implementing fully integrated or total EHR systems. Data were collected over 7 months. A total of 1200 survey questionnaires were distributed, and Alpha Hospital exhibited the highest usable responses (40%), followed by Gamma Hospital (36%) and Beta Hospital (24%).

### Data analysis technique

IS researchers applied partial least squares-structural equation modeling (PLS-SEM) due to the small sample size, nonnormally distributed data, and formative indicators that are inaccurately modeled in covariance-based structural equation modeling (CB-SEM) [43]. PLS path modeling evaluation permits researchers to identify the most potential factors or determinants in predicting target constructs to extend the present theories. This measure was performed along with the formative measures of system quality that contain different components of technological characteristics [44, 45]. Therefore, it is considered the appropriate statistical method for confirmatory factor analysis (CFA) using SmartPLS 3.2.

## Results

### Descriptive analysis

Eight hundred eighty-eight usable responses from the total distributed 1200 surveys, representing a 74% response rate, were subjected to descriptive analysis in SPSS. Table 1 depicts the profile of the respondents. The sample exhibited unequal representation of male (29%) and female (71%) care providers due to a larger percentage of female nurses, specialists, and medical officers in the surveyed hospitals. There was an unbalanced number of respondents who were nurses (44%) out of the total number of respondents due to large recruitment of nurses and shortage of medical officers (doctors) and specialists in MOH hospitals [46] that limits the selection of sample quota for this convenience sampling, despite the confidentiality of population information.

**Table 1:** Characteristics of the study population

	Frequency (*n*)	Percentage (%)
*Hospital*		
Alpha (iSOFT System)	353	40
Beta (F1S1C1EN® System)	213	24
Gamma (Cerner System)	322	36
*Gender*		
Male	256	29
Female	632	71
*Age group*		
< 25	121	14
25–35	565	64
36–45	145	16
46–55	47	5
> 55	10	1
*Education level*		
Diploma	467	53
Bachelor degree	350	39
Master’s degree	65	7
Doctoral degree/Ph.D.	6	1
*Clinical position*		
Assistant Medical Officer	96	11
Medical Officer	328	37
Specialist	71	8
Nurse	393	44
*Year of practice*		
< 5	468	53
5–10	231	26
11–20	142	16
21–30	38	4
> 30	9	1
*Year of EHR system use experience*		
< 3	472	53
3–5	200	23
6–8	120	14
9–11	78	9
> 11	18	2

Approximately 64% of the respondents were aged between 25 and 35 (64%) who were nurses and junior medical officers (housemen). More than half of the respondents were nurses (44%) and assistant medical officers (11%) who had a diploma qualification (53%). In contrast, the medical officers (37%) consisted of those with a bachelor’s or specialist degree (8%), a master’s degree (7%), and a doctoral degree (1%). Many of them (53%) had less than 5 years of practice with less than 3 years of experience using an EHR system. They were considered active EHR system users for less than 3 years because they were junior assistant medical officers, medical officers/doctors, and nurses who were required to perform major tasks with the systems from data entry of clinical documentation to reporting of test results compared to those doctors of more than 10 years of clinical practice and specialists who performed fewer tasks with the systems of than to review, confirm, and validate the patients’ diagnosis and treatment entered by the juniors.

### Common method bias

A common method bias (CMB) was assessed to identify whether the measuring latent constructs explained more than 50% of the variance [47]. Using Harman’s one-factor test, the results demonstrated that the total variance explained was 32.6%, indicating that CMB did not exist in the collected data. Subsequently, a measured latent marker variable (MLMV) method was performed to detect CMB using PLS as suggested by Chin et al. [48]. In the model, a CMB control or marker construct measured by five “attitude towards using technology” items (unrelated to the study construct measures) was added to each exogenous construct. Table 2 displays the results before (original estimates) and after adding a CMB control (MLMV estimates). Changes in path coefficients and *t*-values in the original PLS estimates and MLMV estimates were minimal and not significant, confirming that CMB was not an issue in this study.

**Table 2:** Comparison of path coefficients and t-values by MLMV and original PLS estimation

Relationships	Original estimates (path coefficients)	MLMV estimates (path coefficients)	Original estimates (*t-*value)	MLMV estimates (*t*-value)
System quality → health care provider performance	0.137	0.135	3.526***	3.496***
Records quality → health care provider performance	0.132	0.134	3.492***	3.501***
Service quality → health care provider performance	0.137	0.137	4.573***	4.576***
Knowledge quality → health care provider performance	0.485	0.485	12.598***	12.507***
Effective use → health care provider performance	0.104	0.104	4.346***	4.211***

**1.96 (sig. level = 5%); ***2.57 (sig. level = 1%)

### Formative measurement model analysis

In the hypothesized model, system quality is measured by adequate IT infrastructure, system interoperability, perceived security concerns, and system compatibility. These formative components are represented by indicators that do not highly correlate [44, 49]. For instance, IT infrastructure (required computer hardware, software, and EHR system) is different from an interoperable system (connectivity and workability of different integrated systems). Perceived security concerns are also different from the system compatibility with care providers' clinical tasks. In this study, the formative model was first assessed using a collinearity test. However, the results showed that the score of variance inflation factor (VIF) for every formative indicator or item did not reach the critical level of 5, thus confirming that collinearity was not a significant issue [50].

The assessment continued with the significance and contributions of formative indicators using the bootstrapping feature (with 5000 subsamples) [44, 49]. The results exhibit that all the system quality indicators are scored higher than (*t*-value = 1.96) and significant at a level of 1% (*p* < 0.01), thereby confirming the validity of system quality components and formative measurement model.

### Reflective measurement model analysis

The analysis proceeded with a reflective model assessment in PLS-SEM. As shown in Table 3, the factor loadings for most reflective indicators were higher than a standard of 0.7 to achieve item reliability, except for three indicators, which were still acceptable [45]. Unfortunately, the knowqual_4 indicator with a low loading of 0.542 was removed to improve composite reliability (CR) and average variance extracted (AVE) for its measuring construct. Furthermore, each latent construct's CR and AVE exceeded the suggested thresholds of 0.7 for CR and 0.5 for AVE [45], establishing a convergent validity for the reflective measures.

**Table 3:** Convergent validity

Latent construct	Indicator	Loadings	CR	AVE
Records quality	recqual_1	0.725	0.873	0.535
	recqual_2	0.657		
	recqual_3	0.780		
	recqual_4	0.783		
	recqual_5	0.739		
	recqual_6	0.697		
Service quality	servqual_1	0.834	0.901	0.694
	servqual_2	0.852		
	servqual_3	0.834		
	servqual_4	0.811		
Knowledge quality	knowqual_1	0.826	0.919	0.654
	knowqual_2	0.817		
	knowqual_3	0.858		
	knowqual_5	0.746		
	knowqual_6	0.803		
	knowqual_7	0.799		
Effective use	effuse_1	0.677	0.846	0.649
	effuse_2	0.873		
	effuse_3	0.853		
Health care provider performance	hcperf_1	0.818	0.907	0.709
	hcperf_2	0.819		
	hcperf_3	0.891		
	hcperf_4	0.838		

Discriminant validity for the reflective measures was subsequently assessed by the mean of the Heterotrait–Monotrait Ratio of Correlations (HTMT) criterion [49]. This new standard provides the most conservative threshold of 0.85 for the reflective measures, and the bootstrap confidence intervals must not reach 1 (HTMT < 1) for the statistical inference [49]. As tabulated in Table 4, no value of correlations above 0.85 was recorded. No upper bound of the confidence interval (CI) for every latent construct was recorded as above 1, confirming that a discriminant validity had been established, thus validating the reflective measurement model.

**Table 4:** Discriminant validity

Latent construct	Effective use	Health care provider performance	Knowledge quality	Records quality
Health care provider performance	0.570 CI [0.642]			
Knowledge quality	0.473 CI [0.551]	0.838 CI [0.882]		
Records quality	0.554 CI [0.631]	0.715 CI [0.769]	0.659 CI [0.715]	
Service quality	0.354 CI [0.446]	0.590 CI [0.662]	0.549 CI [0.621]	0.447 CI [0.526]

*CI* Confidence interval

### Path model analysis

Evaluation of the PLS path model began with the coefficients of determination (*R*^2^) for the predictive accuracy assessment. The estimated *R*^2^ score was 0.641, accounting for 64% of the final target construct variance. Health care provider performance was explained by the four quality constructs and effective use, which is interpreted as marginally substantial with higher predictive power [51] in IT acceptance and success.

**Fig. 2:**
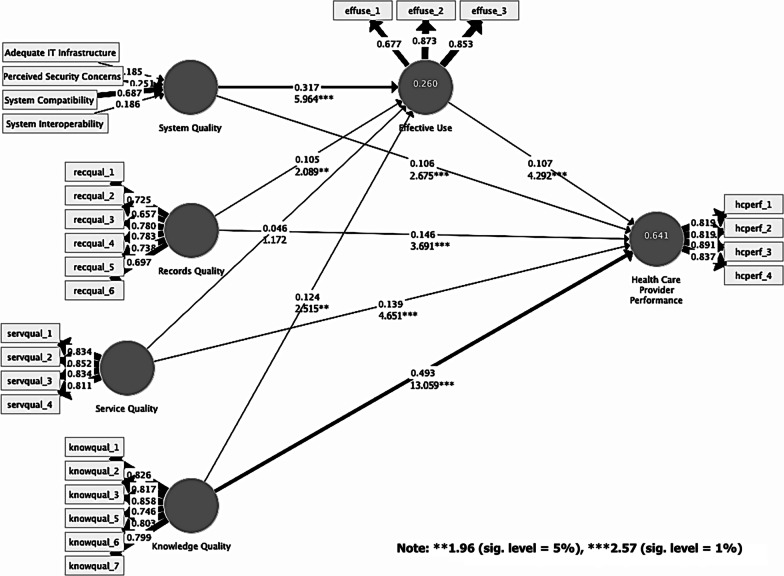
PLS path model

The second step was to assess the path relationships' significance among the latent constructs to validate the hypotheses. Again, using a complete bootstrapping of 5000 subsamples for the two-tailed tests with no sign changes, the hypothesis tests were executed. Figure 2 illustrates the path coefficient scores, *t*-values, and *R*^2^ scores in the path model. Evaluation of this path model entailed five latent constructs to test nine hypothesized relationships and effects. Results revealed that all paths were statistically significant, except for service quality and effective use effects. In other words, hypotheses H1_a_, H1_b_, H2_a_, H2_b_, H3_b_, H4_a_, H4_b_, and H5 were supported when their individual effect scores were equivalent or higher than (*t-*value = 2.57) with significance level at 1% (*p* < 0.01), or equivalent or higher than (*t*-value = 1.96) with significance level at 5% (*p* < 0.05). System quality was the highest predictor for effective use (path coefficient = 0.317, *t*-value = 5.964), while knowledge quality exhibited the largest path coefficient (0.493) and positive effect (*t*-value = 13.059) on the final target construct.

### Effect size assessment

Path model evaluation continued with assessing effect size (*f*^2^) for every study construct over its measuring target construct. In particular, knowledge quality has a large effect size on user performance (*f*^2^ = 0.370) followed by service quality (*f*^2^ = 0.040, small effect), records quality (*f*^2^ = 0.025, small effect), effective use (*f*^2^ = 0.024, small effect), and system quality (*f*^2^ = 0.012, no effect) [52] These results verify the significant contribution of quality of clinical knowledge learnt from EHR systems in predicting the care providers’ performance.

## Discussion

This study determined that system quality is the most crucial construct influencing the effective use of an EHR system. An EHR system can simultaneously perform patient care and simultaneously record diagnosis results if compatible with the CPGs and clinicians' workflows [26]. If treatment notes are available in a user-friendly template, they enable an easy data entry process, allowing more time for doctors and nurses to communicate with their patients. A user-oriented CIS design enables efficient use through automatic data checking and filtering, along with timely access [38]. Furthermore, a system interface design that shows patients' full medical histories can support meaningful use [53].

System quality was also found to affect user performance positively. Results indicated that the structure and content of the systems were compatible with the care providers' working styles. The flow of the systems was designed to fit the different care delivery methods by clinicians after many change requests were updated. As a result, the system use will reduce care providers' workloads from minimal data entry and documentation works, which in turn increases task productivity. Correspondingly, a cross-sectional survey found that ease of use and HIS efficiency positively affected job satisfaction and care providers' work performance in southern Taiwan hospitals [17]. Similarly, an online survey with 219 California residents indicated that system quality, information quality, and service quality measures positively affected physicians' work impact [18].

Record quality was also found to influence effective use positively. A standardized, user-friendly EHR format enables speedy and reduced data entry for the care providers to perform timely diagnosis and treatment without delays. The standardized EHR also (a) improves the consistency of medical records creation among clinics and (b) supports the referral process across other hospitals. Using the autocomplete feature, the doctors can provide the right prescriptions to the pharmacist without making spelling errors. Adopting a critical care system was also found to positively influence doctors' and nurses' acceptance by improving the quality of records and system access and decreasing data entry [7, 30]. Successful clinical system adoption relies on ease of access, completeness, correctness, and standardized EHRs [38]. High-quality EHRs will significantly improve the efficiency of care and administration of medication by nurses [36].

Record quality was also found to have a positive effect on the performance of end-users. EHRs store complete patient medical histories extracted from their treatment notes, images, laboratory results, prescriptions, referral activities, and discharge summaries to facilitate coordinated care among clinics and hospitals from patient birth to death. Therefore, instant access to patient EHRs is critical for their responsible care providers to immediately understand past care, allergies, medications, and patients' follow-ups. Doctors generally do not know about the health status of a patient during his or her first visit. Full and timely access to EHR will avoid further delays. The providers can deliver the best treatment or transfer care, if necessary, without misdiagnosis, repeated tests, wasted resources, or inaccurate medications, increasing their performance. The current finding is thus in line with prior studies on EHR adoption. These studies found that the use of EHR had a positive effect on the physicians' tasks in an intensive care unit by allowing more time to be spent on clinical review with multiple physicians simultaneously and less time performing documentation and administrative work [31]. As indicated in a previous study, the use of EHR also enhances nursing communication skills when interacting and recording patient medical records [54].

The relationship between service quality and effective use was positive but nonsignificant, signifying user dissatisfaction with IT technical support quality. This finding might be due to the frustration of several care providers with the delays in vendor service support following problems with the system or computer. The most frequent technical issues were reportedly related to the low performance of hardware caused by obsolete computers and servers, which complicated the support for an increasing number of system users. System performance was typically slow during the peak hours in the afternoon when the hospitals received many patient visits. A few unplanned system downtimes were triggered by damaged network switches, forcing the users to use paper-based records. As indicated in a previous study, an insignificant impact is typically triggered by low technical service quality, such as incompetent staff, inadequate computers, unplanned/frequent network breakdown, and power interruption [55]. Continuous IT service support for EHR systems, computers, and networks, as well as effective end-user training, are indeed the core determinants for accelerating EHR system adoption [21].

In contrast, service quality was found to influence the performance of health care providers positively. This effect may be attributed to the system vendors' efficient follow-up activities, which ensured that user-reported problems were fully resolved. If the problems were related to the operating system, then the help desk support would troubleshoot the problem via a remote desktop. In hardware malfunction, the help desk support would send their staff to the actual location to fix the issue. A follow-up call would be made after a few hours to verify that the problems had been completely solved so that the users can perform their jobs with greater satisfaction. Hence, immediate support and approachable staff were concluded to significantly influence service quality, improving clinician productivity through timely patient care [17].

Knowledge quality positively affected the effective use and exhibited the most substantial positive effect on performance among the estimated relationships. Doctors who made the right clinical decisions after reviewing the EHRs performed timely and best care, improving effective use. Besides, housemen can learn past patient care of similar conditions provided by specialists with longer practice. These specialists may improve their medical practices through shared treatment with senior doctors. Experienced clinicians can write a more detailed radiology report compared to their less-experienced juniors. Different specialists with different specialties will record every clinical procedure in the EHRs shared and enhanced by other responsible doctors. The systems used to consult patient records, results, and reports can also create and disseminate new medical knowledge for efficient problem solving and decision-making by various care providers. The quality of care will increase to the highest standard and positively affect the care providers' productivity by fully exploring this knowledge. Past research has proven that knowledge quality is positively affected by knowledge management system benefits, system usage, and user satisfaction [34]. Therefore, individual and organizational learning in a health institution must be developed by fostering knowledge creation, storage, and sharing via an EHR system among medical personnel [17].

The effective use of an EHR system was found to influence the performance of care providers positively. The respondents agreed that they could accomplish their clinical tasks in simple steps. The benefits of usage empowered the care providers to perform timely and accurate care without misdiagnosis or prescribing the wrong medications [56]. As noted, the benefits include ease of search and retrieval of past medical records of patients, well-structured and customized EHRs, fewer documentation errors, and convenience of use within the hospital facility. This result is consistent with those found in previous empirical studies. HIS effective use was found to save time for task completion, cut clinical expenses, and enhance caregivers' productivity with minimal medical error [57, 58]. As a result, a boost in performance is anticipated due to greater satisfaction and task productivity, leading to increased patient loyalty and hospital reputation [59].

This study is not without limitations. However, the improved model did not account for some critical factors that could further explain the health care providers’ outcome to guide the hospitals in developing better performance measurement strategies. The health care setting is complex and dynamic, and implementation science would be more impactful in achieving EHR post-implementation success. For instance, the Consolidated Framework For Implementation Research (CFIR) contained 39 constructs arranged into five key domains (intervention characteristics, outer setting, inner setting, characteristics of individuals, and process) linked to the performance outcomes [60], pointed to several relevant constructs not included in the evaluation. This might be great future work as the present model is constrained. Nonprobability sampling, which was employed for data collection, can restrict the findings' generalizability across other IT hospitals with different complexities of interoperable EHR systems, packages, and modules.

Moreover, sample recruitment was focused on CME and CNE programs attended by voluntary health care providers. Only a few specialists participated in these programs due to their hectic schedules. Additionally, half of the respondents (53%) consisted primarily of nurses and graduated medical officers with less than 5 years of practice.

## Conclusions

This study provides two significant contributions to the present theories and methodology that lead to post-implementation success. First, this study produced a validated questionnaire survey on the performance measurement of a user of an EHR system. Second, this study extended the conventional D&M models by incorporating a new knowledge quality predictor and enhanced effective system use, which was found to contribute to multiple healthcare providers' greater task performance in a mandatory setting. PLS is recommended for predicting different clinicians' performance when using multiple EHR systems, particularly in terms of quality of systems, records, technical support service, knowledge, and effective use acquired from larger samples from the primary health care providers. This study supplies theoretical implications by confirming the quality of new knowledge and improved effective use construct were established as significant predictors and determinants for enhancing the performance of multiple primary care providers in three government hospitals with different systems. Future health informatics researchers can consider these two constructs to evaluate any EHR system's success after implementation.

Practical implications of the findings are also established. The survey instrument can serve as a diagnostic tool that IT hospitals can readily use with multiple-system packages to assess the level of EHR system–user performance of specialists, medical officers, and nurses during adoption, particularly when resistance or negative effects on clinical tasks are reported post utilization. Additionally, EHR system vendors can refer to the validated instrument to investigate causes of specific problems when assessing a new system implementation, such as poor implementation and low adoption rate. For increased efficacy, the system should be further customized to allow the head of clinical departments or system moderator to upload and adjust clinician workloads in compliance with new published CPGs. To increase clinicians' productivity, knowledge quality and effective use requirements should be integrated into an EHR system design and future upgrades. All three systems must be upgraded to the latest version of the data mining feature so that health care providers can quickly learn hospital population, health patterns, and disease trends systematically and efficiently for the early prevention of adverse patient conditions and complications, improving their productivity. Increased productivity of health personnel enhances public loyalty towards the government health care system, contributing to the effectiveness of EHR systems. HIS effectiveness that has received substantial investments enhances patient care and safety. Policymakers at the ministry level should design pay-for-performance programs, such as monetary incentives and certificates of appreciation, for EHR champions, and research grants to support additional medical researchers. The MOH Malaysia might consider integrating system quality, records quality, service quality, knowledge quality, and effective use for future strategic planning associated with implementing an upgraded or a new EHR system.

To capture high and low adoption outcomes, future researchers can integrate the improved model with CFIR by selecting appropriate constructs from intervention, outer setting, structural, and individual characteristics tailored to the local hospital context. A comprehensive evaluation using larger samples from different clinical specialties is, therefore, recommended. A longitudinal study with mixed methods can further strengthen the understanding and explanation of which factors predict implementation success measured by health care providers’ performance to determine EHR champions. Integration of both frameworks will develop a more practical model for future researchers and the best CPG for care providers.

## Supplementary Information

**Additional file 1**. Survey Questionnaire.

## Data Availability

The datasets used and analyzed during the current study are available from the corresponding author on reasonable request.

## Abbreviations

AVE: Average variance extracted
CFA: Confirmatory factor analysis
CFIR: Consolidated framework for implementation research
CMB: Common method bias
CME: Continuing medical education
CNE: Continuing nursing education
CPG: Clinical practice guideline
CR: Composite Reliability
EHR: Electronic health record
EMR: Electronic medical record
HIS: Hospital/health information system
HTMT: Heterotrait–monotrait ratio of correlations
KMS: Knowledge management system
MOH: Ministry of Health
MREC: Medical Research and Ethics Committee
PLS-SEM: Partial least squares-structural equation modeling
SPSS: Statistical package for the social sciences

## References

[1] Waegemann C. Ehr vs. cpr vs. emr. Healthcare Informatics online; 2003.

[2] Salleh MIM, Abdullah R, Zakaria N. Electronic health records’ system characteristics, use, and effectiveness: A proposed theoretical framework. In: Soliman KS, editor. Proceedings of the 24th International Business Information Management Association conference. Milan, Italy: International Business Information Management Association (IBIMA); 2014. p. 1669–81.

[3] ISO/TC (2005). ISO/TR 20514:2005(E): health informatics-electronic health record-definition, scope and context.

[4] Abdullah ZS (2013). Hospital information systems implementation framework: critical success factors for Malaysian public hospitals.

[5] Salleh MI, Zakaria N, Abdullah R (2016). The influence of system quality characteristics on health care providers’ performance: empirical evidence from Malaysia. J Infect Public Health.

[6] Liebovitz D (2015). Next steps for electronic health records to improve the diagnostic process. Diagnosis.

[7] Yusof MM (2015). A case study evaluation of a critical care information system adoption using the socio-technical and fit approach. Int J Med Inform.

[8] Salahuddin L, Ismail Z, Hashim UR, Raja Ikram RR, Ismail NH (2019). Mohayat MHN@: sociotechnical factors influencing unsafe use of hospital information systems: a qualitative study in Malaysian government hospitals. Health Inform J.

[9] Ahmed EAS, Ahmad MN, Othman SH (2016). Health information system critical success factors (HISCFs): a systematic literature review. J Inf Syst Res Innov.

[10] Ahmadi H, Nilashi M, Shahmoradi L, Ibrahim O (2017). Hospital information system adoption: expert perspectives on an adoption framework for Malaysian public hospitals. Comput Hum Behav.

[11] Mohamadali NA, Zahari NA (2017). The organization factors as barrier for sustainable health information systems (HIS)—a review. Procedia Comput Sci.

[12] Salleh MIM, Abdullah R, Zakaria N, Comite U (2017). Extending health information system evaluation with an importance-performance map analysis. Advances in health management.

[13] Wei LH, Thurasamy R (2018). An examination of the effects of task technology fit and hospital information system satisfaction in Public Hospital Malaysia: a structural model. Adv Sci Lett.

[14] Ahmadi H, Nilashi M, Shahmoradi L, Ibrahim O, Sadoughi F, Alizadeh M, Alizadeh A (2018). The moderating effect of hospital size on inter and intra-organizational factors of Hospital Information System adoption. Technol Forecast Soc Change.

[15] DeLone W, McLean E (1992). Information systems success: the quest for the dependent variable. Inf Syst Res.

[16] DeLone WH, McLean ER (2003). The DeLone and McLean model of information systems success: a ten-year update. J Manag Inf Syst.

[17] Chang I-C, Li Y-C, Wu T-Y, Yen DC (2012). Electronic medical record quality and its impact on user satisfaction—healthcare providers’ point of view. Gov Inf Q.

[18] Li F (2014). A framework for examining relationships among electronic health record (EHR) system design, implementation, physicians’ work impact.

[19] McGill T, Hobbs V, Klobas J (2003). User developed applications and information systems success: a test of DeLone and McLean’s model. Inf Resour Manag J.

[20] Sedera D, Tan F. User satisfaction: an overarching measure of enterprise system success. In: Pacific Asia conference on information systems. Bangkok, Thailand: Association for Information Systems; 2005. p. 963–76.

[21] Nguyen L, Bellucci E, Nguyen LT (2014). Electronic health records implementation: an evaluation of information system impact and contingency factors. Int J Med Inform.

[22] Häyrinen K, Saranto K, Nykänen P (2008). Definition, structure, content, use and impacts of electronic health records: a review of the research literature. Int J Med Inform.

[23] Fairbanks M (2012). Electronic health record in rural health and alternative health information technology infrastructure models.

[24] Gray C (2014). Electronic health record systems in a centralized computing services environment: critical success factors for implementation.

[25] Rankin P (2016). Interoperability in an inefficient legislative system. Consult Pharm.

[26] Baillieu R, Hoang H, Sripipatana A, Nair S, Lin SC (2020). Impact of health information technology optimization on clinical quality performance in health centers: a national cross-sectional study. PLoS ONE.

[27] Chang C-S, Chen S-Y, Lan Y-T (2012). Motivating medical information system performance by system quality, service quality, and job satisfaction for evidence-based practice. BMC Med Inform Decis Mak.

[28] Schiza EC, Fakas GJ, Pattichis CS, Petkov N, Schizas CN. Data protection issues of integrated electronic health records (EHR). In: XIV Mediterranean conference on medical and biological engineering and computing 2016. Paphos, Cyprus: Springer; 2016. p. 781–4.

[29] Dishaw M, Strong D, Bandy D. Extending the task-technology fit model with self-efficacy constructs. In: Americas conference on information systems 2002 proceedings; 2002. p. 1021–7.

[30] Schreier G. Nurses’ viewpoints on barriers and facilitators to use hospital information systems. In: Schreier G, Hayn D, Eggerth A, editors. dHealth 2020-biomedical informatics for health and care: proceedings of the 14th health informatics meets digital health conference, vol 271. Amsterdam, Netherlands: IOS Press BV; 2020. p. 145–52. 10.3233/SHTI200090 32578557

[31] Carayon P, Wetterneck TB, Alyousef B, Brown RL, Cartmill RS, McGuire K, Hoonakker PLT, Slagle J, Van Roy KS, Walker JM, Weinger MB, Xie A, Wood KE (2015). Impact of electronic health record technology on the work and workflow of physicians in the intensive care unit. Int J Med Inform.

[32] Hung SY, Tsai JCA, Chuang CC (2014). Investigating primary health care nurses’ intention to use information technology: an empirical study in Taiwan. Decis Support Syst.

[33] Boas SJ, Bishop TF, Ryan AM, Shih SC, Casalino LP (2014). Electronic health records and technical assistance to improve quality of primary care: lessons for regional extension centers. Healthcare.

[34] Wu J-H, Wang Y-M (2006). Measuring KMS success: a respecification of the DeLone and McLean’s model. Inf Manag.

[35] Tsai JC-A, Hung S-Y (2016). Determinants of knowledge management system adoption in healthcare. J Organ Comput Electron Commer.

[36] Tajirian T, Stergiopoulos V, Strudwick G, Sequeira L, Sanches M, Kemp J, Ramamoorthi K, Zhang T, Jankowicz D (2020). The influence of electronic health record use on physician burnout: cross-sectional survey. J Med Internet Res.

[37] Paré G, Raymond L, de Guinea AO, Poba-Nzaou P, Trudel M-C, Marsan J, Micheneau T (2015). Electronic health record usage behaviors in primary care medical practices: a survey of family physicians in Canada. Int J Med Inform.

[38] Hoerbst A, Schweitzer M (2015). A systematic investigation on barriers and critical success factors for clinical information systems in integrated care settings. Yearb Med Inform.

[39] Redd TK, Doberne JW, Lattin D, Yackel TR, Eriksson CO, Mohan V, Gold JA, Ash JS, Chiang MF. Variability in electronic health record usage and perceptions among specialty vs. primary care physicians. In: AMIA annual symposium proceedings. American Medical Informatics Association; 2015. p. 2053–62. PMC4765603 26958305

[40] Field A (2013). Discovering statistics using IBM SPSS statistics.

[41] Faul F, Erdfelder E, Buchner A, Lang A-G (2009). Statistical power analyses using G*Power 3.1: tests for correlation and regression analyses. Behav Res Methods.

[42] Sulaiman H, Wickramasinghe N (2014). Assimilating healthcare information systems in a Malaysian hospital. Commun Assoc Inf Syst.

[43] Hair JF, Sarstedt M, Pieper TM, Ringle CM (2012). The use of partial least squares structural equation modeling in strategic management research: a review of past practices and recommendations for future applications. Long Range Plan.

[44] Hair JF, Hult GTM, Ringle C, Sarstedt M (2014). A primer on partial least squares structural equation modeling (PLS-SEM).

[45] Hair JF, Sarstedt M, Ringle CM, Gudergan SP (2017). Advanced issues in partial least squares structural equation modeling.

[46] Ministry of Health Malaysia (2016). Pelan Strategik KKM 2016–2020.

[47] Akter S, D’Ambra J, Ray P (2011). Trustworthiness in mHealth information services: an assessment of a hierarchical model with mediating and moderating effects using partial least squares (PLS). J Am Soc Inf Sci Technol.

[48] Chin WW, Thatcher JB, Wright RT, Steel D, Abdi H, Chin WW, Vinzi VE, Russolillo G, Trinchera L (2013). Controlling for common method variance in PLS analysis: the measured latent marker variable approach. Springer proceedings in mathematics & statistics.

[49] Hair JF, Hult GTM, Ringle CM, Sarstedt M (2017). A primer on partial least squares structural equation modeling (PLS-SEM).

[50] Ringle C, Sarstedt M, Straub D (2012). A critical look at the use of PLS-SEM in MIS quarterly. MIS Q.

[51] Chin WW, Marcoulides GA (1998). The partial least squares approach to structural equation modeling. Modern methods for business research.

[52] Cohen J (2013). Statistical power analysis for the behavioral sciences.

[53] Jensen LGL, Bossen C (2016). Factors affecting physicians’ use of a dedicated overview interface in an electronic health record: the importance of standard information and standard. Int J Med Inform.

[54] Palumbo MV, Sandoval M, Hart V, Drill C (2016). Teaching electronic health record communication skills. CIN Comput Inform Nurs.

[55] Tilahun B, Fritz F (2015). Comprehensive evaluation of electronic medical record system use and user satisfaction at five low-resource setting hospitals in Ethiopia. JMIR Med Inform.

[56] Classen DC, Holmgren AJ, Newmark LP, Seger D, Danforth M, Bates DW (2020). National trends in the safety performance of electronic health record systems from 2009 to 2018. JAMA Netw Open.

[57] Sultan F, Aziz MT, Khokhar I, Qadri H, Abbas M, Mukhtar A, Manzoor W, Yusuf MA (2014). Development of an in-house hospital information system in a hospital in Pakistan. Int J Med Inform.

[58] Hunt S, Chakraborty J. Dose verification errors in hospitals: literature review of the eMAR-based systems used by nurses. J Nurs Care Qual. 2020. 10.1097/NCQ.0000000000000491 10.1097/NCQ.0000000000000491 32541426

[59] Bossen C, Jensen LG, Udsen FW (2013). Evaluation of a comprehensive EHR based on the DeLone and McLean model for IS success: approach, results, and success factors. Int J Med Inform.

[60] Damschroder LJ, Aron DC, Keith RE, Kirsh SR, Alexander JA, Lowery JC (2009). Fostering implementation of health services research findings into practice: a consolidated framework for advancing implementation science. Implement Sci.

