# Supplementary material for: Evaluating the effects of electronic health records system adoption on the performance of Malaysian health care providers
Source: BMC Med Inform Decis Mak. 2021 Feb 25;21:75. doi: 10.1186/s12911-021-01447-4 (PMC7908801; doi:10.1186/s12911-021-01447-4)
Supplement: Supplementary file 1 — Additional file 1. Survey Questionnaire. [file 12911_2021_1447_MOESM1_ESM.docx]

**Appendix I: Revised Survey Questionnaire**

| **Study Construct** | **Component** | **Adopted Item/ Related Studies** | **Indicator/ Improved Item** |
| --- | --- | --- | --- |
| System Quality | *Adequate IT Infrastructure* | My facility is equipped with faster network access [26]. | sysqual_1: Faster network access is critical for me to use EHR system. |
|  |  | My facility is equipped with adequate, up to date, and reliable computer hardware [26]. | sysqual_2: Adequate computer hardware is critical for me to use EHR system. |
|  | *System Interoperability* |  | sysqual_3: I only need to enter and save data once, then use the system with multiple EHR interfaces  (Self-designed). |
|  |  | The overall cost for patient’s treatment is reduced with EHR system interoperability ([68]. | sysqual_4: The cost for patient’s treatment is reduced with the use of EHR system. |
|  |  | The interoperability of different EHR systems is critical to enable a coordinated patient care (Self-designed). | sysqual_5: The connection between different EHR systems is critical to enable coordinated patient care. |
|  | *Perceived Security Concerns* | I believe that the security system does not allow unauthorized access [69]. | sysqual_6: I believe my EHR system does not allow unauthorized access. |
|  |  | I believe EHR system protects patient’s information  (Self-designed). | sysqual_7: I believe my EHR system protects patient’s information. |
|  |  | I believe EHR system has a robust security control and protocol  (Self-designed). | sysqual_8: I believe my EHR system has a robust security control. |
|  |  |  | sysqual_9: I feel secure and safe using EHR system  (Self-designed). |
|  | *System Compatibility* |  | sysqual_10: EHR system fits my workflows  (Self-designed). |
|  |  | The system fits the way I work and my work styles [70]. | sysqual_11: EHR system fits the way I work and my work styles. |
|  |  | The system fits my practice preferences [70]. | sysqual_12: EHR system fits my clinical practices. |
|  |  | The system fits my service needs [70]. | sysqual_13: EHR system fits my patients’ needs. |
| Records Quality | | The information output is timely and up-to-date [71]. | recqual_1: Access to EHRs is timely. |
|  |  | The information output is consistent when sharing patient records [71] | recqual_2: EHRs are consistent when viewing from other computers. |
|  |  |  | recqual_3: EHRs are available in a standardized format  (Self-designed). |
|  |  | The information output is accurate and reliable [71]. | recqual_4: EHRs are accurate. |
|  |  | The information output is complete [71]. | recqual_5: EHRs are complete. |
|  |  |  | recqual_6: EHRs avoid duplication of diagnostic tests  (Self-designed). |
| Service Quality | | The IT support personnel provide prompt service to users [18]. | servqual_1: IT support staff/vendor provides quick assistance when I face problems with EHR system. |
|  |  |  | servqual_2: IT support staff/vendor is always able to solve my problems with EHR system  (Self-designed). |
|  |  | The IT support personnel provide follow-up service to users [18]. | servqual_3: IT support staff/vendor provides follow-up service to EHR system users like me. |
|  |  | The IT support personnel provide adequate training, which allows me to use EHR system  (Self-designed). | servqual_4: IT support staff/vendor provides adequate training for me to use EHR system. |
| Knowledge Quality | | The system is beneficial for learning new knowledge [19, 24]. | knowqual_1: EHR system is useful for learning new medical knowledge. |
|  |  | The system is beneficial to researching or inventing useful knowledge [19, 24]. | knowqual_2: EHR system is useful when researching or creating new medical knowledge. |
|  |  | The system is beneficial to applying knowledge to work [19, 24]. | knowqual_3: EHR system is helpful when applying medical knowledge to my tasks. |
|  |  | The system assists me to share my knowledge [19, 24]. | knowqual_4: EHR system helps me share my medical knowledge with others. |
|  |  |  | knowqual_5: EHR system provides knowledge that increases my ability to make clinical decisions  (Self-designed). |
|  |  |  | knowqual_6: EHR system provides knowledge that improves my ability to solve clinical problems (Self-designed). |
|  |  | The system provides a complete knowledge portal in that I can link to other knowledge sources for more inquiries [19, 24]. | knowqual_7: EHR system provides a complete medical source that I can refer to for more information. |
| Effective Use | |  | effuse_1: EHR system enables me to complete my tasks successfully in a few easy steps  (Self-designed). |
|  |  |  | effuse_2: EHR system allows me to prevent misdiagnosis  (Self-designed). |
|  |  |  | effuse_3: EHR system allows me to provide the right medications to patients  (Self-designed). |
| Health Care Provider Performance | |  | hcperf_1: EHR system increases my time with patients  (Self-designed). |
|  |  |  | hcperf_2: EHR system enhances the safety of patient care  (Self-designed). |
|  |  | The system increases my job productivity [71]. | hcperf_3: EHR system increases my work productivity. |
|  |  |  | hcperf_4: EHR system increases my chances of obtaining better annual performance marks  (Self-designed). |
